# Can postfertile life stages evolve as an anticancer mechanism?

**DOI:** 10.1371/journal.pbio.3000565

**Published:** 2019-12-05

**Authors:** Frédéric Thomas, Mathieu Giraudeau, François Renaud, Beata Ujvari, Benjamin Roche, Pascal Pujol, Michel Raymond, Jean-François Lemaitre, Alexandra Alvergne

**Affiliations:** 1 Centre de Recherches Ecologiques et Evolutives sur le Cancer/Centre de Recherches en Ecologie et Evolution de la Santé, Unité Mixte de Recherches, Institut de Recherches pour le Développement 224-Centre National de la Recherche Scientifique 5290-Université de Montpellier, Montpellier, France; 2 Centre for Integrative Ecology, School of Life and Environmental Sciences, Deakin University, Waurn Ponds, Victoria, Australia; 3 School of Natural Sciences, University of Tasmania, Hobart, Tasmania, Australia; 4 Unité mixte internationale de Modélisation Mathématique et Informatique des Systèmes Complexes, Unité Mixte de Recherches, Institut de Recherches pour le développement/Sorbonne Université, France; 5 Departamento de Etología, Fauna Silvestre y Animales de Laboratorio, Facultad de Medicina Veterinaria y Zootecnia, Universidad Nacional Autónoma de México (UNAM), Ciudad de México, México; 6 CHU Arnaud de Villeneuve, Montpellier, France; 7 ISEM, Université de Montpellier, CNRS, IRD, EPHE, Montpellier, France; 8 Centre National de la Recherche Scientifique, Unité mixte de recherche 5558, Laboratoire de Biométrie et Biologie Evolutive, Université Lyon 1 Villeurbanne, France; 9 Institute of Social and Cultural Anthropology, School of Anthropology and Museum Ethnography, University of Oxford, United Kingdom

## Abstract

Why a postfertile stage has evolved in females of some species has puzzled evolutionary biologists for over 50 years. We propose that existing adaptive explanations have underestimated in their formulation an important parameter operating both at the specific and the individual levels: the balance between cancer risks and cancer defenses. During their life, most multicellular organisms naturally accumulate oncogenic processes in their body. In parallel, reproduction, notably the pregnancy process in mammals, exacerbates the progression of existing tumors in females. When, for various ecological or evolutionary reasons, anticancer defenses are too weak, given cancer risk, older females could not pursue their reproduction without triggering fatal metastatic cancers, nor even maintain a normal reproductive physiology if the latter also promotes the growth of existing oncogenic processes, e.g., hormone-dependent malignancies. At least until stronger anticancer defenses are selected for in these species, females could achieve higher inclusive fitness by ceasing their reproduction and/or going through menopause (assuming that these traits are easier to select than anticancer defenses), thereby limiting the risk of premature death due to metastatic cancers. Because relatively few species experience such an evolutionary mismatch between anticancer defenses and cancer risks, the evolution of prolonged life after reproduction could also be a rare, potentially transient, anticancer adaptation in the animal kingdom.

## Abstract 2

Why menopause evolved in the females of some species remains enigmatic. This Essay proposes that existing adaptive explanations have underestimated an important factor operating both at the specific and the individual levels: the balance between cancer risks and cancer defences.

In several animal species, females cease to reproduce before the end of their natural life span [1]. This occurrence of postfertile life span (Fig 1) has been intensively discussed in mammals, and so far, analyses of demographic data have revealed that females experience a postfertile life stage (in which they outlive their last reproductive events by decades) in humans and in 3 species of toothed whales [2,3]. In these species, postfertile life spans also include menopause, which is an irreversible loss of the physiological capacity to produce offspring due to the permanent cessation of ovulation [4]. Conversely, in other long-lived social mammals (e.g., elephants, blue whales), reproductive life span has extended commensurate with life span [5]. Despite extensive research over the last half-century, the reasons behind the evolution of prolonged postfertile life span and its distribution in the animal kingdom remain at the heart of a continuing debate [6].

**Fig 1:**
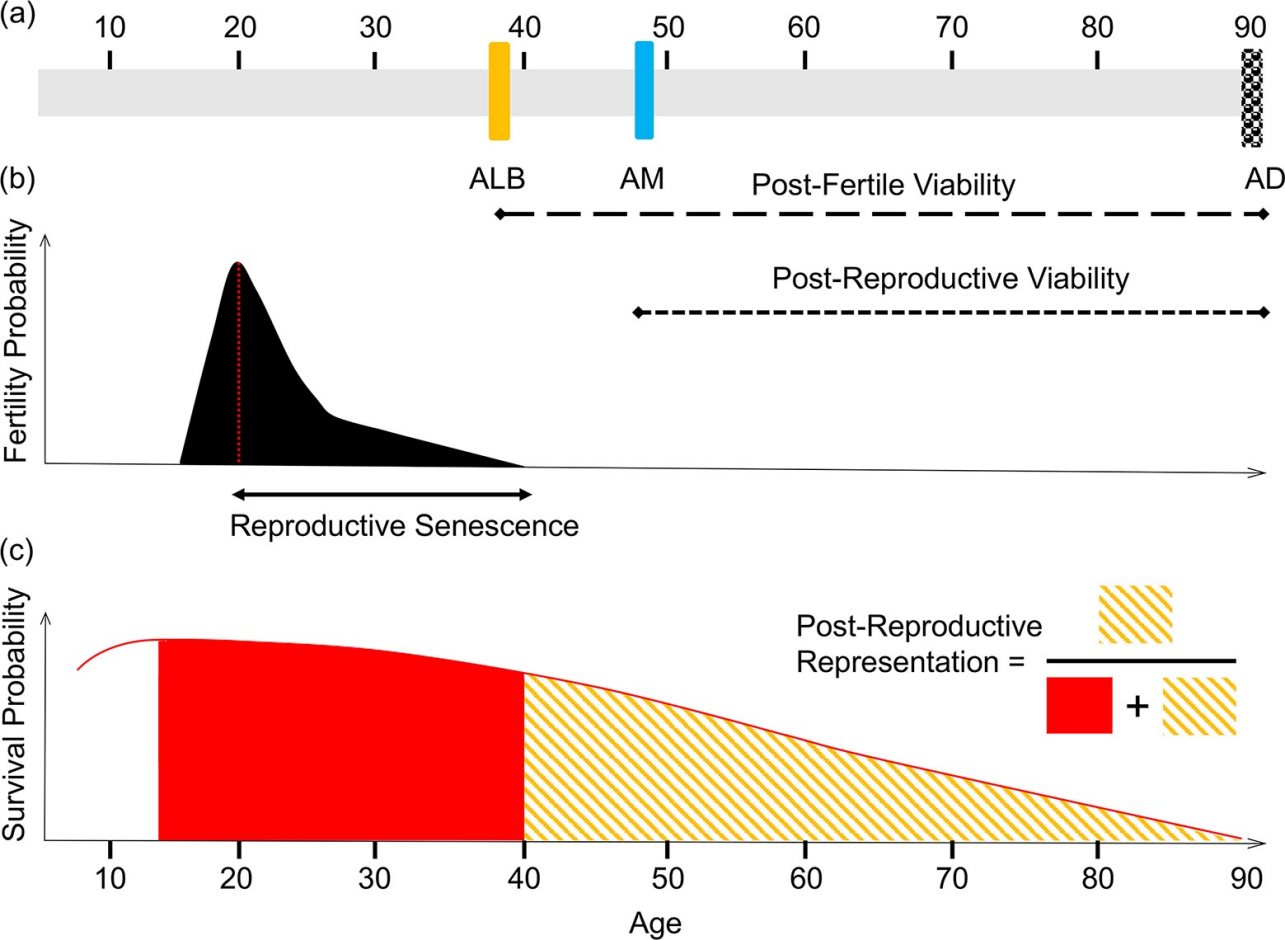
Measuring postreproductive life span: The differences between postfertile viability, postreproductive viability, reproductive senescence, and a postfertile life stage. (A) A woman’s hypothetical life span. Postfertile viability is defined as the length of time between ALB, which typically occurs between 39 and 41 years (reviewed in [69]) and AD. By contrast, postreproductive viability is defined as the length of time between AM and AD. (B) Reproductive senescence. Reproductive senescence corresponds to fertility decline over age, which culminates in the cessation of fertility (ALB). (C) Postreproductive representation. The extent to which a species displays a postreproductive life stage is informed by the ratio of postfertile adult years lived relative to the total adult years lived. For the sake of simplicity, the age at the onset of actuarial senescence was set at the age at first reproduction [7]. AD, age at death; ALB, age at last birth; AM, age at menopause.

Among the most prominent (but still debated) hypotheses that have been put forward to explain this phenomenon (see [7–14]), the “mother hypothesis” suggests that a postfertile life span has evolved because it protects females from increasing age-related maternal mortality risks, which indirectly protects her existing dependent offspring from a likely death if the mother perishes [15]. Another (and probably the most popular explanation for reproductive cessation) is the “grandmother hypothesis.” It posits that a long postfertile life span in females improves the likelihood that their grandchildren (who carry a quarter of their genes on average) reach the age of sexual maturity. Thus, by helping their relatives to survive and reproduce, perhaps through the transmission of cultural knowledge [16], postreproductive females may increase their inclusive fitness through kin selection [17–19]. Adaptive hypotheses proposed to explain the evolution of postfertile life span converge toward the idea that reproduction through time progressively compromises the health and survival prospects of females (e.g., [20] [21], see also [22] for a synthesis in women), which ultimately jeopardizes the fate of their offspring and grandoffspring, as observed in Asian elephants, *Elephas maximus* [23,24]. Several kinds of pregnancy-related complications and health problems have indeed been identified in aging women (e.g., [25,26]), although it is unclear whether this is a derived condition [19]. Here, we contend that a crucial parameter underpinning the evolution of postreproductive fertile life span has been largely overlooked so far: the balance between cancer risks and cancer defenses.

There are several reasons why we believe that this balance might have influenced the evolution of an extended postfertile life span:

1 Oncogenic processes are ubiquitous, and their abundances increase with age. Metazoans have been living with cancer since the origins of multicellularity [27] and, in return, have developed various cancer defenses [28]. However, these adaptations often keep oncogenic progressions under control without necessarily eradicating them [29,30]. Not surprisingly, oncogenic manifestations are highly prevalent in host populations, and it is thus a normal phenomenon that all individuals harbor and accumulate precancerous lesions and in situ tumors during their life in a variety of organs (e.g., prostate, lung, thyroid, breast, pancreas) (see [31–37]), even if they do not necessarily lead to metastatic cancers.

Whereas cancer incidence increases with age in Western populations [38] (with, however, a plateau or a decline at very old ages [39]), cancer seems to be a rare occurrence in populations facing conditions more closely resembling those during which postfertile life span might have evolved (i.e., high fertility and high mortality). The few data collected among hunter–gatherer populations suggest that cancer incidence is low, in particular, with respect to breast, endometrial, and ovarian cancers (among the Tsimane [40]) or colon cancers (among the Inuits [41]), cancers known to be associated with a Western lifestyle (higher exposure to reproductive hormones due to lower and delayed fertility, rich diet, etc) [42]. However, women living in traditional populations are likely to face a risk of cancer from infectious diseases, as indeed about 20% of cancers are caused by pathogens [43,44]. Reproductive tract infections represent 47.3% (43.7–51.0) among women living in the rural Gambia [45], and among the Guarani women of Argentina, pap smears show an inflammatory pattern for 96% of patients, with a possible infectious agent found in 58% of cases [46]. In Papua New Guinea, 59% of reproductive-aged women present a sexually transmitted infection [47]. Finally, among Dai women living in rural South China, human papillomavirus infection prevalence is the highest in the older age group (>56 years) [48]. More data in traditional populations are needed to evaluate how cancer risk increases with age and pregnancy in women.

2 Parity (the number of pregnancies reaching viable gestational stage, including live births and stillbirth) promotes the growth of existing tumors. Data from Western populations show that parity has a dual effect on breast cancer risk [49]. Full-term pregnancies, especially when they occur in early life (<30 years), decrease breast cancer risk in the long term [50–52]. However, in the short term, pregnancy also transiently increases cancer risk, because it boosts the development of oncogene-activated cells into tumors and/or promotes a metastatic cascade [53,54]. The most common malignant proliferations occurring during pregnancy include malignant melanoma, malignant lymphomas, and leukemia, as well as cervix, breast, ovary, colon, and thyroid cancers [55, 56]. The highest risk occurs in the first 5 years after giving birth, and parous women remain at an increased risk of breast cancer for more than 20 years as compared with nulliparous women [57]. Reasons behind this higher risk are multiple, including the local suppression of the adaptive immune system, especially during the first trimester when the mother’s cell-mediated immunity is strategically suppressed to allow for successful implantation [58]. This period is also characterized by relatively high inflammatory status, which is necessary for implantation [54,59]. Hormonal changes, permeability, and vascularization are also involved in the pathophysiology of cancer associated with pregnancy [60,61].
3 Reproductive cessation and/or menopause could prevent a metastatic cascade. In light of the two aforementioned considerations, we propose that reproductive cessation in females has evolved because after a given age, pregnancy would be associated with a higher probability of premature death due to invasive cancers (Fig 2). This is because boosting the growth of an already large number of existing tumors (see previous) would increase the probability of tipping the balance toward the initiation of metastatic, uncontrollable cancers. In addition to pregnancy itself, the normal physiology of fertile women is also expected to promote the growth of tumors, notably, cancers that are dependent on hormones for growth and/or survival. For instance, fluctuating levels of circulating estrogen and progesterone during the menstrual cycle are known to increase breast cancer susceptibility in women (e.g., estrogen receptor and breast cancer [62,63], see also [64] for a recent review, but see also [65,66]). Menopause (i.e., the cessation of menstrual bleeding following a 12-month period of amenorrhea [67,68]) typically occurs between 45 and 55 years of age in women [69] and may have thus evolved as a “natural hormonal therapy” to stop, or limit, the growth of such kind of malignancies before a fatal threshold is reached (see also [70]).

**Fig 2:**
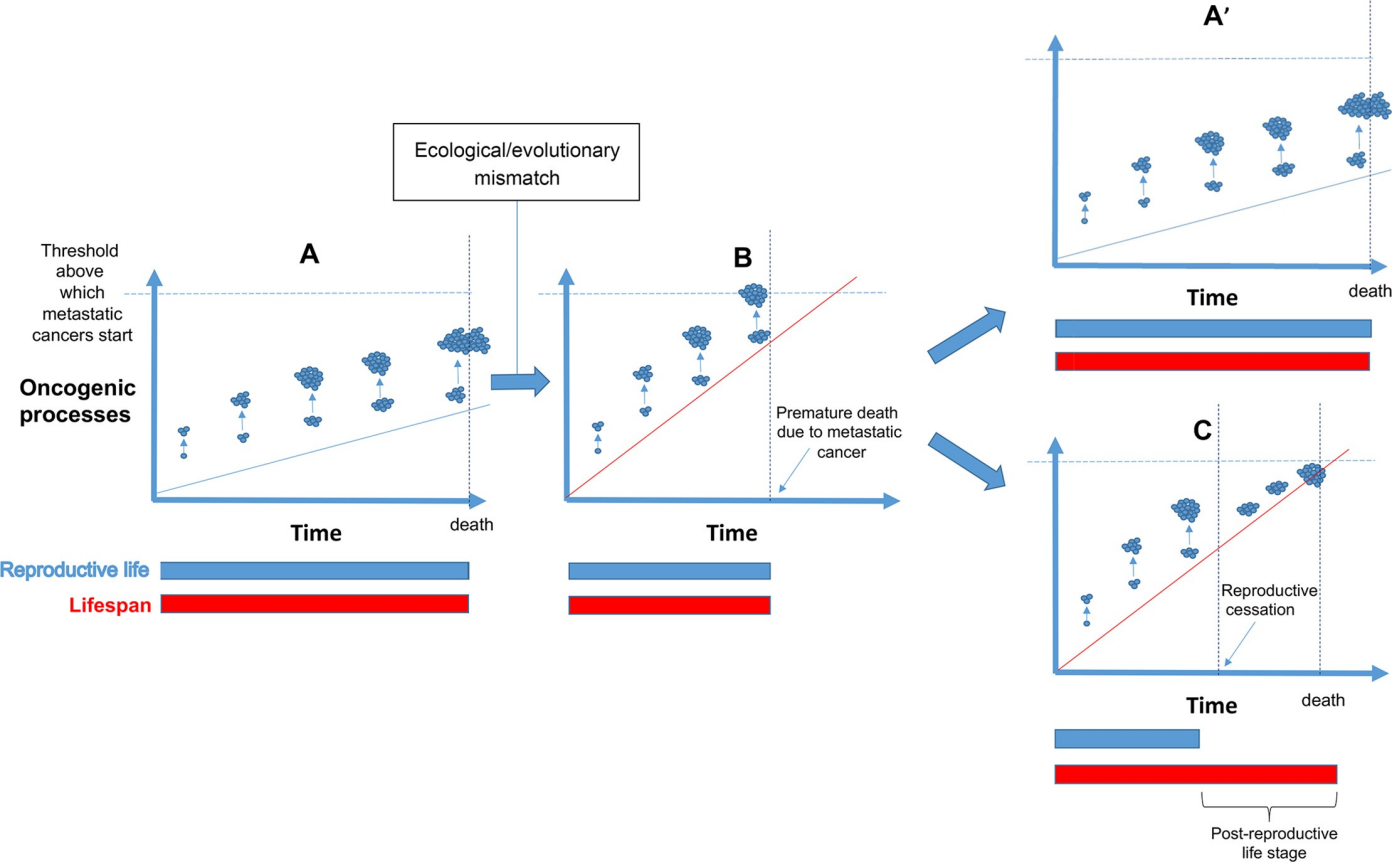
Evolution of postreproductive life stage in relationship with the balance between cancer defense and cancer risk. (**A**) In species possessing cancer defenses in alignment with cancer risks, oncogenic lesions only slowly accumulate through time (blue line). Even if reproductive episodes exacerbate the growth of existing tumors (blue circles), they are not sufficient to induce metastatic cancers: reproduction occurs throughout the life span, and the fitness is maximal. (**B)** When cancer defenses are too weak, given cancer risks (because of ecological and/or evolutionary mismatches), oncogenic processes rapidly accumulate (red line), and reproductive episodes can prematurely induce metastatic cancers in aging females, with a short life span and a low fitness as a result. Natural selection can (1) favor in these species the evolution of stronger cancer defenses, yielding again to a situation comparable to (**A**), here (**A’**), but also (2) favor females ceasing their reproduction prematurely to preserve their health (**C)**. In that case, females’ fitness is higher than in (**B**) because a post-reproductive stage permits grandparental care, which enhances inclusive fitness. The (**C**) scenario can be just a transient situation until additional cancer defenses are selected and bring back the species to the (**A/A’**) situation.

The existence of a postfertile life span in hominids is not a recent phenomenon. Since hominin longevity exceeded 50 years of age more than 1 million years ago [71], postfertile and postreproductive life spans (Fig 1) probably existed in early *Homo erectus* and *H*. *ergaster* [72]. The same thinking applies to early *H*. *sapiens*, as 17% of prehistoric foragers among them apparently survived beyond age 40 years [73]. In addition, although estimates vary between studies, one-third of foragers in traditional populations without easy access to modern medicine lived beyond age 40 years [74]. Interestingly, epidemiological studies (e.g., [40,75]) suggest that some reproductive cancers (ovarian, breast, prostate, and endometrium) are rare in traditional populations compared with industrialized ones, which is in accordance with our hypothesis. Indeed, reproductive cessation efficiently protects women from cancers in populations having a lifestyle close to the one experienced thousands of years ago, whereas it becomes insufficient for women from industrialized countries, recently exposed to novel evolutionary mismatches exacerbating cancer risks (e.g., [76]). Because women outlived their last birth during much of humans’ historical and evolutionary past, it is now challenging to evaluate the fitness benefits (in terms of cancer avoidance) of early reproductive cessation. One indirect way (although imperfect given numerous environmental changes) to assess this benefit would be to quantify cancer risk in postmenopausal women from industrialized countries/Western societies who are receiving hormone replacement therapy (see next).

The reason why the physiological process of menopause typically occurs several years after the last delivery (at least in humans and the short-finned pilot whale, *Globicephala macrorhynchus* [77] [78]) can be explained if the normal physiology of fertile women, although oncogenic because of hormonal exposure, is less oncogenic than pregnancy itself. Avoiding pregnancy early without stopping reproductive capacity would allow individuals to experience a period of time with an attenuated cancer risk. Such a postfertile but premenopausal period might also free individuals from the health issues that are triggered by menopause and associated declining levels of estrogen (e.g., osteoporosis and cardiovascular diseases in Western populations [79–82]; hot flushes in the United States, Europe, and Africa; and depression in Asia, Africa, and Europe [83]), potentially facilitating parental and grandparental care. Generally speaking, the relationship between hormonal levels and menopause symptoms is complex and mediated by sociocultural factors. Although comparative evidence suggests that there are no fewer symptoms among women living in developing populations, it is difficult to ascertain whether contemporary chronic diseases are reflective of past conditions. Still, the possibility that ceasing reproduction first and ceasing reproductive capacity only after could be the best compromise to preserve long-term female health is, as of yet, unexplored.

Several observations seem to support our hypothesis. For instance, it is well established that late pregnancies are indeed associated with a significantly enhanced risk of breast cancer for women [84,85]. Late menopause (i.e., after age 55) is also associated, all else being equal, with an increased risk of ovarian, breast, and uterine cancers, because longer exposure to estrogen increases a woman's risk of cancers [86]. Also, as women go through menopause and experience declining levels of estrogen, they have a significantly reduced breast cancer risk, the intensity of hot flushes being even inversely associated with the risk [87]. Reciprocally, women treated with hormone replacement therapy (HRT) (e.g., estrogen and progestin, a form of artificial progesterone) to relieve menopausal symptoms have an enhanced risk of hormonally dependent cancer, because continued hormone exposure among postmenopausal women promotes the late stages of carcinogenesis and facilitates the proliferation of malignant cells [88,90]. The magnitude of the increase in breast cancer risk per year of hormone use is similar to that associated with delaying menopause by a year [89]. Very recently [91], it has been estimated that 6.3% of women who never used HRT developed breast cancer, compared with 8.3% of women who used the drug continually for 5 years. This study also indicates that the longer women used menopausal hormone therapy, the greater their risk of breast cancer. This higher risk is maintained more than a decade after they have stopped taking the drug. Although this work does not demonstrate that menopausal hormone therapy per se causes breast cancer, it suggests that hormone replacement therapy mediates this risk: using HRT maintains women in a kind of premenopausal state, keeping them from getting the protective benefits of menopause on cancer risk.

Finally, although reproductive cessation could be argued to be a by-product of antagonistic pleiotropy and selection for fertility early in life [92,93], women who naturally possess an enhanced risk of cancer, due to, for instance, germline mutations in human tumor suppressor genes (also known as caretaker genes) BRCA1 and BRCA2, also have an earlier onset of ovarian senescence [94] and an earlier natural menopause compared with unaffected women (on average, 3 years [95]). This suggests that an early menopause could be an evolved adaptation to higher cancer risk. Further studies would, however, be necessary to determine if this potentially evolved mechanism is genetically linked to the BRCA1 or 2 loci and/or results from phenotypic plasticity when an enhanced risk of cancer (e.g., high rate of malignant cell productions) is perceived by the organism.

Although an extended postreproductive life span has been suggested to evolve via kin selection [96,97], other mathematical models [6,97,98] and some empirical evidence in contemporary foragers [8] suggest that the benefit of parental [98] or grandparental [8, 98] care alone [6, 99] is not strong enough to favor the evolution of an extended postfertile life span: a combination of factors is needed [6]. Thus, the “intergenerational reproductive conflict hypothesis” [5,100] has been proposed to account for the evolution of the decoupling between actuarial and reproductive senescence (i.e., the decline in age-specific survival and reproductive probabilities, respectively). This view posits that an extended postreproductive life span can evolve as a consequence of female reproductive competition within families in female-dispersing populations. If paternal grandmothers are genetically related to their daughters-in-law’s children, the reverse is not true. Thus, in the case of a reproductive overlap between women of the same social unit, paternal grandmothers would suffer disproportionate fitness costs as compared with their daughters-in-laws. The model predicts that the costs of reproductive competition combined with the benefits of grandparenting would lead to the evolution of female early reproductive cessation in female-dispersing populations. Although this theory has received empirical support in killer whales [100], studies in humans have reported mixed results, which is partly due to the contrasted environments faced by human populations. A reproductive conflict between in-laws has been shown to impact child survival [101] among historical Finns. In the contemporary Gambia, such a conflict is avoided because mothers-in-law have stopped reproducing when their son’s wives give birth to their first child [102]. By contrast, cobreeding has been linked with fitness benefits, rather than costs, in a historical Norwegian population [11], and reproductive senescence is not accelerated in a female-dispersing population in Indonesia [103]. Thus, to explain the evolution of the unique, long postfertile life span in humans, additional mechanisms are needed. Malignant proliferations could intervene as a selective force because mothers developing tumors are likely to be in a poorer condition than healthy mothers, providing a suboptimal fetal environment (e.g., smaller nutritional intake, chronic inflammation, compromised placental function yielding a poor delivery of nutrients and oxygen to the fetus), with subsequent detrimental consequences for their child health (independently of treatments, e.g., [104]). In addition, several maternal cancers in older women, especially melanoma but also hematopoietic malignancies and lung cancer, have been reported to metastasize from the placenta to the fetus [105,106].

Because oncogenic processes are extremely widespread among metazoans [107], one might be surprised (if our hypothesis is correct) to see that despite extensive evidence of reproductive senescence (decline in the reproductive system as one aspect of general degenerative aging processes, e.g., age-specific decrease in the size of sexual traits linked to fertilization efficiency and in the quality and quantity of parental care) [108], reproductive cessation and especially menopause remain rare in the animal kingdom. Following the logic of our hypothesis, these traits should be selected for as a life-history adaptation to cancer in species that do not otherwise possess defenses that are efficient enough against malignant proliferation, given the size and longevity of organisms and/or their level of exposure to mutagenic substances. Different processes can lead to this situation. First, let’s recall that cancer itself exerts a selective pressure that has shaped numerous adaptations in multicellular organisms, either preventing the formation of neoplasms or controlling their growth and progression [109]. Broadly, cancer defense mechanisms fall into two categories: (1) the ability to eradicate tumors and (2) the capacity to limit the proliferative potential of neoplasms [28]. However, it is also increasingly suggested that individuals bearing tumors might adjust their life-history strategy, for instance, by breeding at a younger age or by investing more in reproduction to compensate for a potential decrease of fitness caused by the development of malignancies (e.g., in *Drosophila* [110] and Tasmanian devils (*Sarcophilus harrisii*) [111]). There is thus no conceptual obstacle to cancer being a selective pressure shaping late life-history traits (e.g., reproductive senescence and cessation, menopause) as well.

In parallel, several evolutionary processes can generate a mismatch between cancer defenses and cancer risks. Genetic drift and inbreeding processes can, for instance, exacerbate malignant problems [112]. Also, species that, for any reason, have recently underwent a change of size and/or longevity could be at a higher risk of cancer. Leroi and colleagues [113] predicted that cancer selection (to prevent or alleviate fitness costs due to cancer) should be especially important as animals evolve new morphologies or larger, longer-lived bodies. Because this selection necessarily takes more or less time depending on contexts [114], some species can transiently experience a mismatch between their cancer risks and their level of defenses. As a typical example, the increased incidence of bone cancer in larger dogs compared with smaller ones [114] suggests that, during the domestication process, selection on genes responsible for higher stature (and hence an enhanced number of cells) has not been accompanied by selection for more efficient cancer defenses (e.g., [115–117]). Humans are another typical example. Even if data are undoubtedly much less accurate in nonhuman animals, our lifetime risk of developing malignant cancer in Western populations (38.4%) is one of the highest in the animal kingdom [115,118,119]. Although recent mismatches (abundance of food, tobacco, alcohol, lack of physical activity, etc) undoubtedly explain a significant part of these statistics, we cannot exclude that there is a significant contribution of older mismatches for which only relatively imperfect solutions have been until now retained by selection. Interestingly, as compared with other species, humans have also experienced major and recent evolutionary changes in their anatomy, physiology, and life history, especially longevity, in which the proportion of individuals surviving to old ages has increased since the Early Upper Paleolithic (e.g., [120–124]). It has also been suggested that an extended life span in females could result from a by-product of selection for longevity in males [125] and/or because younger females prefer older males [126]. Certain authors (e.g., [1]), however, argue that these processes cannot explain why long-lived females cease reproduction long before death. Our hypothesis could solve this enigma because an increasing amount of evidence (e.g., [119,127]) suggests that humans possess cancer defenses that are too weak given the cancer risks they have incurred following their recent evolution. Evolutionary mismatches in our species also undoubtedly occurred each time we modified our environmental conditions (e.g., sanitation, healthcare, reduced predation, increase in food availability). Until additional effective cancer defenses are selected for in species exhibiting an evolutionary mismatch between cancer risk and their cancer defenses (see [28]), it is expected that aging females will experience higher cancer risks, with reproductive cessation and menopause being a transient way to reduce fitness costs induced by oncogenic processes.

The extent to which the 3 species of toothed whales that experience postreproductive life span are more like humans than other long-lived mammals like elephants and blue whales is unclear, although some speculation remains possible. The long-finned pilot whale (*G*. *melas*) and short-finned pilot whale (*G*. *macrorhynchus*) are closely related species. However, although cancers have been reported in both species [128], only the latter experiences reproductive cessation. *G*. *macrorhynchus* is also much bigger (i.e., with more cells) (800 kg versus 1,000–3,000 kg) suggesting (and assuming that cancer is only limited by the occurrence of oncogenic mutations, but see [54]) that it could potentially be, in the absence of supplementary cancer defenses as compared with *G*. *melas*, at a higher risk of cancer. For killer whales (*Orcinus orca*), who are huge predators, a large size is likely to be a trait favored by natural selection, as it permits the hunting of large preys. Contrary to other large and/or long-lived animals (e.g., other whales, elephants, naked mole rats) for which it has been recently demonstrated that they possess remarkable anticancer defenses [115,129,130], killer whales may not possess fully effective anticancer defenses given their size. Recently, detailed postmortem analyses of ovarian activities in toothed whales suggest that a postreproductive life span might also have evolved in beluga whales (*Delphinapterus leucas*) and narwhals (*Monodon monoceros*) [77]. These recent case studies provide an indirect support for our hypothesis because beluga are large and vulnerable to cancer (as illustrated by their propensity to easily convert environmental pollution into malignant pathologies [131]) and narwhals display a low level of genetic diversity [132], which could indirectly exacerbate cancer risk [112]. The genetics of narwhals indicates that populations have grown rapidly since the start of the last glacial period around 115,000 years ago but before this had been slowly declining for about a million years [133]. This low genetic diversity level is therefore not recent, suggesting that compensatory adaptations to cancer risks may have been selected.

Among primates, females show a unique pattern of extended postreproductive life span. Indeed, detailed analyses of 7 primate populations longitudinally monitored have revealed that the number of females experiencing a postreproductive life was particularly low in nonhuman primates (from 1% in baboons, *Papio cynocephalus* to 6% in muriquis, *Brachyteles hypoxanthus*), as compared with the population of human foragers considered in the study (42.5% in the !Kung population) [133]. In light of our hypothesis, this finding appears particularly striking and deserves explanations. Tumors are common in primates [107], and these species also show clear evidence of reproductive senescence at both the demographic (e.g., [133]) and physiological (e.g., [134]) levels. As natural condition likely shapes reproductive senescence patterns across species [135], one might expect that the female rate of reproductive senescence is fastened when the risk of getting cancer increases disproportionately with the age-specific reproductive allocation, especially in primates in which offspring survival and condition are enhanced by the presence of their grandmother (e.g., vervet monkeys, *Chlorocebus aethiops sabaeu*s, [136]). However, even in a species like the Japanese macaque (*Macaca fuscata*), in which the presence of a grandmother increases juvenile survival [137], the proportion of females experiencing a postreproductive life span remains extremely low [2]. Taken together, these results suggest that cancer defense mechanisms are sufficiently efficient in nonhuman primates to align reproductive life span with the mortality pattern of aging individuals. This assumption is also supported by recent studies showing that nonhuman primates, contrary to humans, possess tumor suppression systems that function through life (e.g., a reduced requirement for the primate-specific adrenal androgen-mediated kill switches tumor suppression system, e.g., [119]). Thus, although nonhuman primates also accumulate various kinds of genomic damage, the subclass of oncogenic processes able to initiate tumorigenesis is efficiently extinguished. Because it is uncommon in nature to observe organisms displaying evolutionary mismatches, it could also explain why reproductive cessation and menopause are so rare in primates and, more generally, across the entire animal kingdom.

There are several possibilities for testing our hypothesis empirically, both within and across species. Whenever we refer to “reproductive schedule,” we envision several traits: skewed reproduction (i.e., the front loading of reproductive events and stopping reproduction early), reproductive senescence patterns, age at last birth, age at menopause, and also the length of the perimenopausal period (cross-culturally, most women describe menopause as a prolonged process rather than a distinct event [81]). Whether the “cancer hypothesis” is better suited to explaining patterns of variation in all or only some of those traits remains to be investigated. Following on from our hypothesis, we tentatively suggest a few predictions, as follows:

1. A postreproductive life stage is not expected to evolve in species with adequate cancer resistance mechanisms. In Asian elephants, the evolution of highly efficient cancer defenses might explain why, despite the presence of mothers and grandmothers enhancing calf survival and reproduction [24], relatively few individuals reach a postreproductive life stage. The absence of selection on postreproductive life span despite its impact on inclusive fitness in those species could be explained by the low prevalence of cancer at all ages (<5% [115]). Elephants are known for their efficient cancer defense mechanisms, including the high number of copies of the tumor suppressor gene tumor protein p53 (*TP53*) in both African and Asian elephants [138, 139] and the accelerated evolution of the DNA repair gene Fanconi anemia complementation group L (*FANCL*) in African elephants [140]. By contrast, in humans, the number of *TP53* copies is limited to 1 (as compared with 20 in African elephants), and at least in killer whales, there is no evidence for an accelerated evolution of the *FANCL* gene following selection for increased body size [140], suggesting inadequate cancer resistance mechanisms (i.e., that reduce fitness) in this species. In order to provide a direct test of our hypothesis, however, a phylogenomics analysis of cancer defense genes in mammal species characterized by extended maternal investment would be needed to disentangle phylogenetics from cancer effects on life-history variation. Similarly, this hypothesis could be explored experimentally, given that the same changes could be engineered in laboratory animals like mice.
2. Cancer selects for the evolution of a postreproductive life stage in species that display both inadequate cancer defenses and parental investment. Previous studies have shown that cancer can accelerate life history in *Drosophila* [110] and Tasmanian devils [111]. However, whether cancer is a strong enough pressure to select for the evolution of reproductive cessation and menopause currently lacks direct empirical evidence. One could conduct artificial selection experiment in 2 closely related mammal species characterized by different levels of maternal investment and manipulate both the efficiency of cancer defenses (e.g., by artificially selecting for longevity without selecting for better DNA repair mechanisms) and cancer risk (e.g., through exposure to pollutants). We predict that increasing exposure to cancer would select for the acceleration of reproductive senescence in species with both maternal investment and inadequate cancer defenses (difference between cancer defenses and cancer exposure) but not (or less so) in others. In theory, cancer could also select for a postreproductive life span in males of species that display both obligate paternal investment (e.g., in titi and owl monkeys) and inadequate cancer defenses.
3. Contrasted cancer risks can select for different patterns of reproductive schedule. If populations present a history of high cancer risk in old ages (e.g., due to recent increases in stature or longevity), selection for the front loading of fertility events and early reproductive cessation is expected. Conversely, if populations present a history of low cancer risk, for instance, because of a diet rich in antioxidants, as is often the case among populations characterized by extreme longevity, selection for late reproduction is expected. This might explain an association reported by a recent study conducted in a population of Sardinian women showing that late last birth increases the probability of becoming centenarian [141]. This association suggests that late births, which might occur because of a slower rate of ovarian aging and/or different cultural norms around age at last birth, are not associated with an increased risk of mortality in women. In this context, the selective pressure to stop reproducing early because of cancer risk is likely to be relaxed as compared with other populations. The extent to which late reproduction among centenarians results from positive selection on specific genetic variants, strategic plastic adjustments to early life, or simply a slow aging process is yet to be investigated.
4. Reproductive senescence protects against cancer. First, genetic polymorphism in cancer resistance is predicted to shape age-specific relationships between pregnancy and cancer progression. For instance, in humans, the *TP53* gene exhibits polymorphism across populations and individuals, with p53 codon 72 polymorphism being associated with the risk of developing gastric, colorectal, and other cancers [142,143]. Whether such polymorphism also correlates with patterns of reproductive senescence (e.g., the age at which pregnancy significantly increases the probability of developing cancer) has not, to our knowledge, been explored to date. Second, for cancer to act as a selective pressure on reproductive senescence, an increase in cancer-related mortality postpregnancy must be at least as important as the increase in mortality due to childbirth or other sources of mortality. This condition is potentially met in social species in which the risk of environmentally driven mortality is buffered [144]. A possibility would be to investigate whether age at last birth is a better predictor of cancer-related mortality relative to all-causes mortality, controlling for parity. A test of this prediction in humans should be conducted in high-fertility subsistence populations facing higher risks of mortality both in infancy and from childbirth and not only in women from Western countries. If the condition is not met, it does not necessarily mean that reproductive cessation does not protect from cancer, but it would suggest that cancer is unlikely to have selected for reproductive cessation.
5. Cancer has favored the evolution of phenotypic plasticity in reproductive schedule. It is not excluded that human phenotypic variation in reproductive senescence could be partly explained by cancer risk determined through development. Indeed, the age-specific cost of pregnancy in terms of cancer progression potentially varies as a function of early life conditions. Telomere length, a cancer defense, is known to be influenced by development. For instance, maternal obesity predicts shorter telomeres among elderly female offspring [145], which suggests that polymorphism in cancer resistance is partly shaped by the early life environment. In addition, telomeres are longer in women with late maternal age, suggesting that polymorphism in cancer resistance in mothers [146], itself partly shaped by the early life environment, predicts variation in maternal age at last birth. There is also evidence for a critical role of the childhood environment in shaping the number of oocytes and the rate of follicular atresia in humans [72]; thus, early life conditions can produce a positive correlation between cancer risk in old ages and the rate of reproductive senescence. Such a positive correlation might be understood as the by-product of an accelerated somatic senescence or an adaptive response to an acceleration of the age at which cancer defenses cannot counteract the impact of pregnancy on cancer progression. Although it has been shown that adverse early conditions lead to the acceleration of reproductive development [147] and, thus, a faster somatic senescence through pleiotropic effects [148], it remains to be investigated whether childhood conditions influence even more strongly the age at which pregnancy promotes cancer progression. Note that the adjustment of reproductive patterns to environmental conditions experienced early in life could be achieved through either biological (i.e., rate of follicular atresia) or cultural mechanisms (i.e., early age at marriage in women, stopping reproduction before menopause).
6. Cancers occur in the longevity gain period provided by sheltered environments. Thanks to protected environmental conditions offered by zoos, most captive mammalian populations show a delayed onset of actuarial senescence and an extended life span compared with their wild counterparts [149]. As a consequence of the uncoupling between actuarial and reproductive senescence [135], extended periods of post-reproductive lifespan are observed in a wider range of captive populations as compared to wild ones [1]. We predict that the occurrence of cancer should be exacerbated during the “longevity gain period” observed in zoos because females having reproduced at their full potential during their reproductive life should display a particularly high risk of cancer (see previous section). In addition, the number of cancers reported within this (artifactitious) postreproductive life span period should be particularly pronounced in primate females showing a reproductive allocation pattern close to the one observed in humans (e.g., Callitrichidae [150]). Everything else being equal, we also predict that this elevated risk of cancer during this longevity gain period should be more pronounced in females than in males and should mostly concern reproductive cancers.

## Concluding remarks

The “cancer hypothesis” proposed here for the evolution of an extended postreproductive life is not, per se, novel nor incompatible with other hypotheses. Because in existing theories, oncogenic-related health problems already fall within the category of factors that could modulate the payoffs of old females reproducing themselves versus investing into existing offspring and grandoffspring. In addition, although neoplasia are common in mammals [107], they are less frequent in insects that can sometimes display menopause (e.g., the aphid glue-bomb [151]). Here, the evolutionary scenario we propose for the evolution of postfertile life spans typically concerns species that already display grandparental care and experience an evolutionary mismatch and for which reproduction favors, at least transiently, malignant proliferation in females. Also, an important assumption in our hypothesis is that it is easier to evolve a postreproductive life span than more effective anticancer defenses. Although cultural mechanisms could explain the rapid spread of early cessation of reproduction, this assumption remains to be investigated for the physiological process of menopause. Nevertheless, we believe that oncogenic processes and, more precisely, the balance between their dynamics in relationship with the level of host defenses could play a major role in the selection of postreproductive life in animals. Future mathematical formalizations are now needed to improve our understanding of the conditions required for the cancer hypothesis to be a realistic scenario. In addition, there is a need for data on how cancer risk (beyond breast cancer risk) increases with age and pregnancy in high-mortality populations following traditional lifestyles: How much does it increase with age? Is cancer risk influenced by pregnancy events? What are the fitness costs associated with postmenopausal diseases? How intense should the selection pressure exerted by cancer risk be? Finally, because ecosystems all over the world are increasingly polluted with mutagenic substances, it is also predicted that numerous species will experience, sooner or later, an evolutionary mismatch between the efficiency of their anticancer defenses and their cancer risks. Further studies would be necessary to determine whether in animal populations (with parental investment) from highly polluted habitats, reproductive cessation in currently evolving as an adaptive trait in females. To conclude, the importance of oncogenic processes for the evolution of life-history traits in changing ecosystems offers promising new research avenues yet to be exploited.
